# Analysis of (CAG)_n_ expansion in *ATXN1*, *ATXN2* and *ATXN3* in Chinese patients with multiple system atrophy

**DOI:** 10.1038/s41598-018-22290-0

**Published:** 2018-03-01

**Authors:** X. Zhou, C. Wang, D. Ding, Z. Chen, Y. Peng, H. Peng, X. Hou, P. Wang, X. Hou, W. Ye, T. Li, H. Yang, R. Qiu, K. Xia, J. Sequeiros, B. Tang, H. Jiang

**Affiliations:** 1 Department of Neurology, Xiangya Hospital, Central South University, Changsha, Hunan 410008 P. R. China; 2 Laboratory of Medical Genetics, Central South University, Changsha, Hunan 410078 P. R. China; 3 Key Laboratory of Hunan Province in Neurodegenerative Disorders, Central South University, Changsha, Hunan 410008 P. R. China; 4 School of Information Science and Engineering, Central South University, Changsha, Hunan 410083 P. R. China; 5 IBMC - Institute for Molecular and Cell Biology, i3S - Instituto de Investigação e Inovação na Saúde; and ICBAS; Univ. Porto, Porto, Portugal; 6 National Clinical Research Center for Geriatric Disorders, Changsha, Hunan 410078 China

## Abstract

Multiple system atrophy (MSA) is a complex and multifactorial neurodegenerative disease, and its pathogenesis remains uncertain. Patients with MSA or spinocerebellar ataxia (SCA) show overlapping clinical phenotypes. Previous studies have reported that intermediate or long CAG expansions in SCA genes have been associated with other neurodegenerative disease. In this study, we screened for the number of CAG repeats in *ATXN1*, 2 and 3 in 200 patients with MSA and 314 healthy controls to evaluate possible associations between (CAG)_n_ in these three polyQ-related genes and MSA. Our findings indicated that longer repeat lengths in *ATXN*2 were associated with increased risk for MSA in Chinese individuals. No relationship was observed between CAG repeat length in the three examined genes and age at onset (AO) of MSA.

## Introduction

Multiple system atrophy (MSA) is an adult-onset neurodegenerative disease characterized by various combinations of autonomic failure, cerebellar ataxia and Parkinsonism^1,2^. MSA is classified into two subtypes, MSA with predominant cerebellar ataxia (MSA-C) and MSA with predominant Parkinsonism (MSA-P)^2^.

Spinocerebellar ataxias (SCAs) are a group of neurodegenerative diseases characterized by cerebellar dysfunction that may be accompanied by other neurological abnormalities^3,4^. Patients with MSA have similar symptoms to those with SCA, including prominent ataxia, dysmetria and eye movement anomalies^3,4^. Several studies have suggested links among neurodegenerative diseases, their causative genes and clinical syndromes characterized by overlapped phenotypes^5–7^. For instance, patients diagnosed with MSA carry expanded CAG repeats in *ATXN3*^8^. Furthermore, research has revealed a strong effect of *ATXN2* expansion on Parkinson disease (PD) and the MSA-P subtype^9^, and intermediate-expansion within *ATXN1* or *ATXN2* has been associated with an increased risk for amyotrophic lateral sclerosis (ALS)^10^. In animal and cell models of ALS, ataxin2 interacts with TDP-43 and forms characteristic cytoplasmic aggregates in neurons, suggesting a relationship between *ATXN2* and TDP-43 toxicity^10,11^.

The specific molecular mechanisms underlying the pathogenesis of MSA remain unclear. However, several genes other than *COQ2* have been implicated in MSA, including *SNCA*, *SHC2* and *ATXN2*^9,12,13^. From this perspective, we aimed to test the hypothesis that CAG repeat sizes for the genes involved in SCA (namely, *ATXN1*, *2* and 3) might play a role as risk factors for MSA and affect age at onset (AO) of the disease.

## Materials and Methods

### Subjects and samples

A total of 200 patients (128 males, 72 females; 35–72 years of age, with a mean age of 53.4 ± 7.6 years) who satisfied consensus criteria for MSA were recruited from the outpatient neurology clinic of Xiangya Hospital, Central South University, Hunan, China^2^. Clinical stage was evaluated using the Unified Multiple System Atrophy Rating Scale (UMSARS). There were 148 patients with MSA-C and 52 patients with MSA-P. This study also included 314 healthy controls with no history of neurodegenerative disease or other diseases.

The control and patient groups were matched with respect to age (age range, 30–74 years; mean age, 53.5 ± 7.6 years), sex ratio (203 males, 111 females), and region of residence. All patients and controls were from the Han Chinese population. The study was approved by the Ethics Committee of Xiangya Hospital, and written informed consent was obtained from all participants.

### DNA analysis and genotype classification

**Figure 1:**
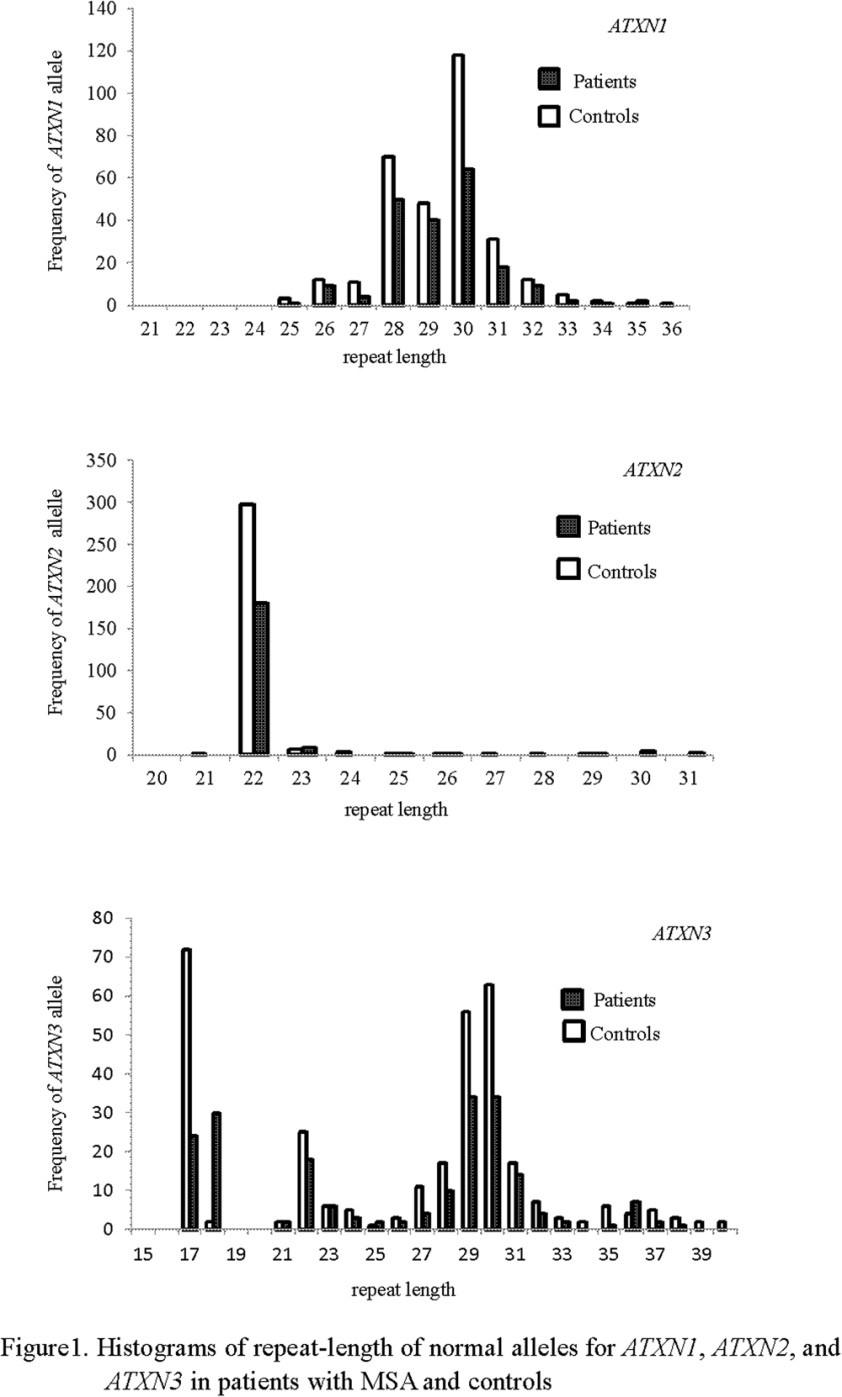
Histograms showing repeat lengths for the long allele in *ATXN1*, *ATXN2*, and *ATXN3* in patients with MSA and controls.

Genomic DNA was extracted from peripheral blood using standard phenol-chloroform extraction procedures^14^. Genotyping of *ATXN1*, 2 and 3 was determined by polymerase chain reaction (PCR) amplification of CAG tracts in combination with capillary electrophoresis, using GeneMarker software (SoftGenetics). At the SCA1, SCA2 and SCA3/MJD loci, the allele containing the larger repeat was designated the ‘long’ allele, and the other allele was regarded as the ‘short’ allele. The short and long alleles were considered separately in statistical models. With respect to the multimodal or skewed distributions in Fig. 1, repeats in long alleles of *ATXN*2 and *ATXN3* were classified as short, short-medium, medium or long in accordance with the approach described in previous studies^15,16^. For *ATXN1*, since the size of the long allele exhibited a nearly normal distribution, we divided long alleles into two groups, short (≤29 repeats) and long (å 29 repeats), based on mean repeat size.

### Statistical analysis

Differences in age and sex between the patients with MSA and the controls were assessed using a t-test and a chi-square test. Descriptive statistics are expressed as mean ± standard deviation (Table 1). Associations between size of (CAG)_n_ and risk for MSA were determined via logistic regression, adjusting for age and sex. We used one-way factorial analysis of variance (ANOVA) or the Kruskal-Wallis test to investigate the association between (CAG)_n_ size and AO for the patients with MSA. The Mann-Whitney U test or t-tests were used to test for differences in repeat length between patients with MSA-C and patients with MSA-P. A two-tailed *P*-value ≤ 0.05 was regarded as significant.

**Table 1:** Demographic data and mean (CAG)_n_ repeats for patients with MSA and controls.

Variable	MSA	Controls
number	200	314
males (%)	128 (64.0)	203 (64.6)
mean age/age at onset ± SD	53.4 ± 7.4	53.5 ± 7.6
age range	35–72	30–74
(CAG)_n_ in the long allele		
*ATXN1:*		
mean ± SD	29.3 ± 1.6	29.4 ± 1.6
range	25–35	25–36
*ATXN2:*		
mean ± SD	22.4 ± 1.6	22.1 ± 0.7
range	22–31	21–29
*ATXN3:*		
mean ± SD	25.6 ± 5.9	26.2 ± 6.1
range	17–38	17–40

*SD* = *standard deviatio*n.

## Results

Table 1 summarizes demographic data for all 514 participants. No pathological (CAG)_n_ expansion in the three SCA genes was detected (in either patients or controls). Distributions of (CAG)_n_ size are shown in Fig. 1. In *ATXN1*, the mean size was 29.3 ± 1.6, ranging 25–35 repeats in patients and 29.4 ± 1.6 in controls, ranging 25–36 repeats. In *ATXN2*, the mean size was 22.4 ± 1.6, ranging 22–31 repeats in patients and in controls 22.1 ± 0.7, ranging 22–29 repeats; 8 patients and 3 controls carried long expansions (Supplementary Table S2). In *ATXN3*, the mean size was 25.6 ± 5.9, ranging 17–38 repeats in patients and in controls 26.2 ± 6.1, ranging 17–40 repeats.

For the *ATXN2* locus, there was a significant difference in the distribution of CAG repeats between patients and controls (*P* = 0.011, OR = 1.253, 95% CI = [1.052–1.492]) (Table 2). The number of CAG repeats in *ATXN1* and *ATXN3* did not significantly differ between patients with MSA and controls. There were no significant correlations between AO for MSA and repeat length in *ATXN1*, *2* or 3 (Table 3). In addition, the distributions of (CAG)_n_ size in any of these three genes did not significantly differ between patients with MSA-C and patients with MSA-P (Supplementary Table S1).

**Table 2:** Comparison of (CAG)_n_ sizes for polyQ-related genes in patients with MSA and controls.

Locus		n	*P*-value	OR	95% CI
*ATXN1*	patients	200	0.793	0.985	0.879–1.104
	controls	314			
*ATXN2*	patients	200	0.011	1.253	1.052–1.492
	controls	314			
*ATXN3*	patients	200	0.207	0.981	0.952–1.011
	controls	314			

*P-values were calculated via logistic regression using SPSS 18*.*0*.

**Table 3:** Effects of (CAG)_n_ size in polyQ-related genes on age at onset for patients with MSA.

Locus	Group (CAGs)	n	Age at onset	*P*-value
*ATXN1*	short	104	52.5 ± 7.2	0.072
	long	96	54.3 ± 7.5	
*ATXN2*	short-medium	181	53.4 ± 7.2	0.842
	medium	11	54.1 ± 8.5	
	long	8	51.6 ± 10.4	
*ATXN3*	short-medium	85	53.3 ± 7.2	0.898
	long	115	53.4 ± 7.5	

^*^Group: Long alleles of *ATXN1*, *ATXN2*, and *ATXN3* were divided into several groups as follows:

*ATXN1*: short: ≤29 CAGs; long: >29 CAGs.

*ATXN2*: short: <22 CAGs; short-medium: 22 CAGs; medium: 23–26 CAGs; long: 27–32 CAGs.

*ATXN3*: short: <19 CAGs; short-medium: 19–25 CAGs; long: 26–40 CAGs.

*P-value estimated by one-way ANOVA or Kruskal-Wallis test*.

## Discussion

None of our patients exhibited pathogenic expansion in any of the three examined polyQ-related genes, indicating that such expansion may not be a causative factor for MSA. Nevertheless, we found a significant association between CAG repeat sizes in *ATXN2* and risk for MSA.

The most common size (over 95%) of the (CAG)_n_ in SCA2 is either 22 or 23 (range, 14–32)^17–20^. Ataxin2, which is encoded by *ATXN2*, is localized to the rough endoplasmic reticulum and plays a critical role in mRNA processing^21^. In the pathogenesis of SCA2, polyQ expansion of ataxin2 confers a gain-of-function mutation that induces neuronal impairment and triggers the disease phenotype^21^. Ataxin2 is also closely related to other neurodegenerative diseases, such as ALS, PD and spinocerebellar ataxia type 3 (SCA3/MJD)^22–25^. Functional studies have proven that ataxin2 interacts with TDP-43 via joint mRNA binding, aggravating TDP-43 toxicity and thereby further increasing the risk of developing ALS^22^. In a yeast model, ataxin2 was shown to be a modifier of α-synuclein biotoxicity in specific molecular pathways and a predictive nodal point in the α-synuclein network^6^. As an mRNA-related translation factor, ataxin2 has been associated with α-synuclein toxicity in neurons of patients with PD^6^. The neuropathological hallmark of MSA is the presence of glial cytoplasmic inclusions (GCIs) containing α-synuclein; given this characteristic, MSA can be regarded as a synucleinopathy, together with PD and Lewy body dementia (DLB)^26–28^. We can speculate that *ATXN2* increases the risk for MSA by perturbing mRNA metabolism and translation and thereby influencing α-synuclein biotoxicity.

An association was found between MSA and CAG repeat sizes in *ATXN2* but not CAG repeat size in *ATXN1* or *ATXN3*, implying that *ATXN2* may play a role as a risk factor for MSA (at least) in the Chinese population; however, no modifying effects of repeat lengths at SCA1, SCA2 or SCA3/MJD loci on AO of MSA were observed, possibly due to sample size. The small number of *ATXN2* long-expansion, over 26 CAGs, found in patients and controls (due to their rarity in the general population) is, of course, a limitation that cannot be easily overcome. Further studies in different ethnic populations and a larger sample size are needed to confirm the present findings.

In terms of mechanisms for neurodegeneration, it would be important to shed some light on a possible pathogenic interaction between ataxin2 and α-synuclein in MSA. The genetic association between *ATXN2* and MSA may contribute to a more comprehensive understanding of neurodegenerative disorders and to foster new therapies for such diseases.

### Ethics statement

This study was approved by the Ethics Committee of Xiangya Hospital of Central South University in China (equivalent to an institutional review board), and all methods were performed in accordance with approved guidelines. Written informed consent was obtained from all participants.

## Electronic supplementary material

Supplementary Information 
