# Supplementary material for: Analysis of (CAG)n expansion in ATXN1, ATXN2 and ATXN3 in Chinese patients with multiple system atrophy
Source: Sci Rep. 2018 Mar 1;8:3889. doi: 10.1038/s41598-018-22290-0 (PMC5832826; doi:10.1038/s41598-018-22290-0)
Supplement: Supplementary file 1 — Supplementary Information [file 41598_2018_22290_MOESM1_ESM.docx]

**Analysis of (CAG)_n_ expansion in *ATXN1*, *ATXN2* and *ATXN3* in Chinese**

**patients with multiple system atrophy**

Xin Zhou ^1^, Chunrong Wang ^1^, Dongxue Ding ^1^, Zhao Chen ^1^, Yun Peng ^1^, Huirong Peng ^1^, Xuan Hou ^1^, Puzhi Wang ^1^, Xiaocan Hou ^1^, Wei Ye ^1^, Tianjiao Li ^1^, Huihua Yang ^1^, Rong Qiu ^4^, Kun Xia ^2^, Jorge Sequeiros ^4^, Beisha Tang ^1,2,^^3^ and Hong Jiang ^1,2,3*^

^1^ Department of Neurology, Xiangya Hospital, Central South University, Changsha, Hunan, 410008, P. R. China.

^2^ State Key Laboratory of Medical Genetics, Central South University, Changsha, Hunan, 410078, P. R. China.

^3^ Key Laboratory of Hunan Province in Neurodegenerative Disorders, Central South University, Changsha, Hunan, 410008, P. R. China

^4^ School of Information Science and Engineering, Central South University, Changsha, Hunan, 410083, P. R. China.

^5^ IBMC - Institute for Molecular and Cell Biology, i3S - Instituto de Investigação e Inovação na Saúde; and ICBAS; Univ. Porto, Portugal.

* Corresponding author:: Dr. Hong Jiang, MD,

Department of Neurology, Xiangya Hospital,

Central South University,

Changsha 410008,

P. R. China

E-mail: jianghong73868@126.com

S1. Comparison of the (CAG)_n_ between patients with MSA-C and MSA-P subtypes

|  |  |  | *ATXN1* |  |  | *ATXN2* |  |  | *ATXN3* |  |
| --- | --- | --- | --- | --- | --- | --- | --- | --- | --- | --- |
|  |  |  | mean | *P*- |  | mean | *P*- |  | mean | *P*- |
|  | n |  | ± SD | value |  | ± SD | value |  | ± SD | value |
| MSA-C | 148 |  | 29.3±1.7 | 0.650 |  | 22.4±1.6 | 0.849 |  | 25.6±5.9 | 0.850 |
| MSA-P | 52 |  | 29.4±1.0 |  |  | 22.4±1.7 |  |  | 25.5±6.0 |  |

SD = standard deviation.

*P*-value was caculated by Mann-Whitney U test or t-test.

S2. Demographic data and repeat sizes of patiens and controls with long CAGs in *ATXN2*

| Group | CAGs in *ATXN2* | Age at onset | Sex | Subtype | Possibility |
| --- | --- | --- | --- | --- | --- |
| case1 | 30 | 55 | female | MSA-P | possible |
| case2 | 31 | 40 | male | MSA-C | probable |
| case3 | 30 | 66 | male | MSA-C | probable |
| case4 | 31 | 56 | female | MSA-P | possible |
| case5 | 30 | 61 | male | MSA-C | possible |
| case6 | 30 | 38 | male | MSA-C | possible |
| case7 | 29 | 42 | female | MSA-C | possible |
| case8 | 30 | 55 | male | MSA-P | probable |
| control1 | 28 | 53 | male |  |  |
| control2 | 27 | 32 | female |  |  |
| control3 | 29 | 55 | male |  |  |
